# A Fluorescent Thermometer Based on a Pyrene-Labeled Thermoresponsive Polymer

**DOI:** 10.3390/s100907979

**Published:** 2010-08-27

**Authors:** Christian Pietsch, Antje Vollrath, Richard Hoogenboom, Ulrich S. Schubert

**Affiliations:** 1 Laboratory of Organic and Macromolecular Chemistry, Friedrich-Schiller-University Jena, Humboldtstrasse 10, 07743 Jena, Germany; E-Mails: christian.pietsch@uni-jena.de (C.P.); antje.vollrath@uni-jena.de (A.V.); ulrich.schubert@uni-jena.de (U.S.S.); 2 Dutch Polymer Institute (DPI), John F. Kennedylaan 2, 5612 AB Eindhoven, Netherlands; 3 Laboratory of Macromolecular Chemistry and Nanoscience, Eindhoven University of Technology, P.O. Box 513, 5600 MB Eindhoven, Netherlands; 4 Supramolecular Chemistry group, Department of Organic Chemistry, Ghent University, Krijgslaan 281 S4, 9000 Ghent, Belgium

**Keywords:** pyrene, DEGMA, stimuli-responsive polymer, lower critical solution temperature (LCST), RAFT polymerization, solvatochromism, fluorescent thermometer

## Abstract

Thermoresponsive polymers that undergo a solubility transition by variation of the temperature are important materials for the development of ‘smart’ materials. In this contribution we exploit the solubility phase transition of poly(methoxy diethylene glycol methacrylate), which is accompanied by a transition from hydrophilic to hydrophobic, for the development of a fluorescent thermometer. To translate the polymer phase transition into a fluorescent response, the polymer was functionalized with pyrene resulting in a change of the emission based on the microenvironment. This approach led to a soluble polymeric fluorescent thermometer with a temperature range from 11 °C to 21 °C. The polymer phase transition that occurs during sensing is studied in detail by dynamic light scattering.

## 1. Introduction

In recent years, dye-functionalization of thermoresponsive polymers has received significant attention for the development of sensory materials [1–12]. This approach allows simple and fast detection of the temperature by measuring the absorbance or fluorescence of a polymer solution. The high sensitivities arise from the incorporated solvatochromic dye molecules [13,14], which respond to minor local environmental changes that occur upon the temperature induced polymer phase transition. Such optical thermometers are desirable for remote sensing of the temperature based on the reversible temperature induced polymer phase transition. These thermometers can be used if the electromagnetic field or the ionic strengths are too strong for a conventional thermometer. A variety of optical polymeric thermometers have been reported as sensing materials. Hirai and co-workers classified these types of responsive polymers in three classes [5,9]: (*i*) heat-induced fluorescence enhancement, (*ii*) heat-induced fluorescence quenching, and (*iii*) selective emission enhancement at a specific temperature range. A further class is based on (*iv*) a temperature-dependent batho-chromic/hypso-chromic shift of the absorption or emission wavelengths [3,6,11,15,16]. Such solvatochromic dyes change color in response to changes of the solvent polarity. Recently, it was reported that combining a solvatochromic dye with a temperature-responsive polymer leads to a color change upon changing the temperature, as in the dissolved state the dye is in contact with water while in the collapsed state the dye is dissolved in the less polar precipitated polymer globule. Such optical polymeric sensors can be used for a wide range of applications, such as biosensors [2], drug delivery [17,18], logic gates [4,10,19,20], and optical sensing [1,5–9,11,12].

The majority of these sensor materials are based on stimuli-responsive polymers, exhibiting a lower critical solution temperature (LCST) in solution, sometimes also called coil-to-globule transition. Such LCST polymers are water-soluble at low temperatures and undergo a sharp entropy-driven collapse with increasing temperature. As a result the temperature sensing regime of LCST-based sensors is often limited to the detection of a narrow temperature range (around 10 °C). The LCST behavior of polymers in solution can be described by the Flory-Huggins theory [21].

Besides the most commonly studied poly(*N*-isopropylacrylamide) (PNIPAM) [17,22], a number of poly(ethyleneglycol) (PEG) based polymers have been reported to exhibit LCST behavior. In particular, ethyleneglycol methyl ether methacrylate-based polymers have attracted attention as thermoresponsive materials and as alternatives to PNIPAM [23–27]. It has been demonstrated that oligoethylene glycol methyl ether methacrylate-based polymers exhibit similar desirable thermoresponsive properties in water as PNIPAM while showing less hysteresis between heating and cooling.

The strong interest in PEG base methacrylates is based on the easy preparation of well-defined structures by controlled radical polymerization (CRP) techniques. In this work we used the reversible addition fragmentation chain transfer (RAFT) technique as polymerization method [28,29]. In particular, we used di(ethylene glycol) methyl ether methacrylate (DEGMA) copolymers, which have a low LCST of around 25 °C and this class of polymers has superb biocompatibility [30,31].

The fluorescent dye pyrene is a very hydrophobic molecule and has limited solubility in polar solvents like water. Pyrene is one of the most studied fluorescent dyes in chemistry. In the early nineties Winnik and co-workers studied the heat-induced phase transition in water of a pyrene functionalized poly(NIPAM) [32,33]. It could be shown that the phase transition has an influence on the pyrene eximer emission. Pyrene is also used in polymer chemistry as fluorescent probe to determine the critical micelle concentration of block copolymers based on the sensitivity of the pyrene emission to the polarity of the solubilizing medium [34,35]. It could be shown that the ratio of the intensity of the pyrene monomer emission (in total five vibronic bands) of the first (I_1_ at 373 nm) and third peak (I_3_ at 384 nm) represents a sensitive parameter, which is characteristic for the polarity of the environment [36]. Also the relative intensity of the first and the fifth peak can be used as sensitive parameter for the polarity of the medium (I_5_ at 393 nm). In addition, pyrene shows a second emission band at higher concentration corresponding to an excited dimer (excimer fluorescence > 450 nm). This excimer fluorescence band appears at higher wavelengths in comparison to the emission band of the monomer fluorescence (red shift) [37].

In the current work, we developed an optical fluorescent temperature sensors with a temperature sensing regime based on the LCST transition of pyrene functionalized poly(DEGMA) in water. The synthesis and characterization of this copolymer are reported. In addition, the temperature sensing ability of the copolymer is discussed based on fluorescence spectroscopy and dynamic light scattering (DLS).

## 2. Results and Discussion

**Scheme 1.**
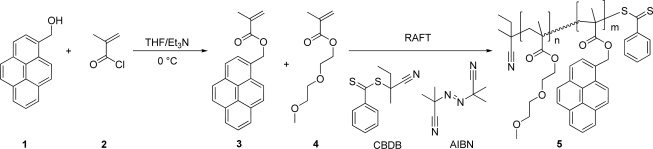
Schematic representation of the synthesis of the pyrene-functionalized monomer and the subsequent RAFT copolymerization with DEGMA.

The synthesis route of the pyrene-labeled copolymer poly(DEGMA-*stat*-PyMMA) (**5**), is depicted in Scheme 1. The polymerization was performed using the RAFT process [28,29] to ensure the preparation of well-defined copolymers allowing a straightforward interpretation of the sensing results. As a first step the polymerizable pyrene dye methacrylate monomer (PyMMA, **3**) was synthesized by an esterification reaction of the hydroxyl group of pyrene-1-methanol (**1**) with methacryloyl chloride (**2**) [12]. In a further step, PyMMA (**3**) was statistically copolymerized with di(ethylene glycol) methylether methacrylate (DEGMA, **4**) by RAFT polymerization. 2-Cyano-2-butyl dithiobenzoate (CBDB) was used as chain transfer agent and azoisobutyronitrile (AIBN) as radical initiator (Scheme 1). The synthesis was performed at 70 °C for 12 hours with toluene as solvent. The monomer to RAFT agent ratio was 100 using 5% of dye-functionalized monomer, aiming for a degree of polymerization of 100.

**Figure 1.**
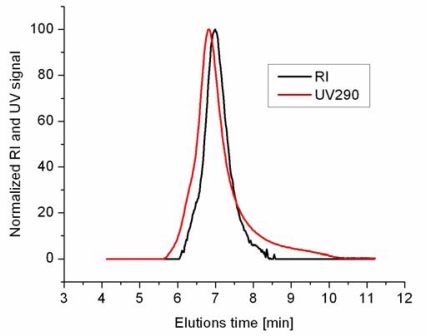
Size exclusion chromatograms of copolymer **5**.

The size exclusion chromatograms display a narrow molar mass distribution with low PDI values (PDI < 1.20). The chromatograms were recorded with both a RI (black line) and a UV detector (red line) revealing nearly the same distribution clearly demonstrating that the pyrene dye is incorporated in the copolymer since PDEGMA does not absorb at 290 nm (Figure 1).

The shift in the retention time between the RI and UV detector is due to the placement of the detectors in series, *i.e.*, the eluent first passes the UV-detector and then the RI detector. The molar mass of copolymer **5** was determined to be 29,000 g/mol, with a polydispersity index of 1.20 calculated for the RI-trace with poly(styrene) standards. A broader molar mass distribution is obtained with the UV detector, which is due to the different sensitivities of both detectors, *i.e.*, higher sensitivity of the UV detector, especially in the oligomer region. In addition, the RI detector detects all repeat units while the UV detector only detects the incorporate pyrene molecules. As such, low molar mass oligomers will not be substantially detected by the RI detector, but when a few of these are dye-labeled they will appear in the UV-detector.

The ratio between the DEGMA units and the pyrene side groups in the copolymer chain was determined to be 4.5 mol% using ^1^H-NMR spectroscopy based on the respective aromatic pyrene signals and the backbone or side-chain signals of the polymer. In addition, the pyrene content was found to be 5.0 mol% based on the UV-vis extinction coefficient of the copolymer. These two values are in good agreement and are within the experimental error of the two applied techniques.

**Figure 2.**
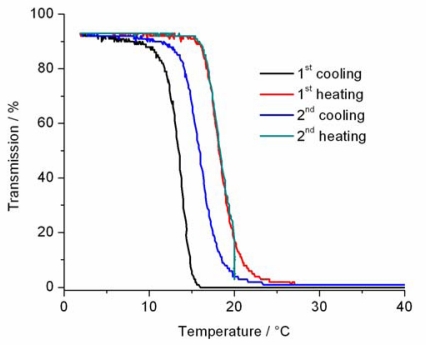
Turbidity *versus* temperature plot for an aqueous solution of copolymer **5** (2.5 mg mL^−1^).

The temperature induced phase transition of the poly(DEGMA-*stat*-PyMMA) copolymer **5** was explored by turbidimetry. Figure 2 shows the change in turbidity (determined at 500 nm) of copolymer **5** in water at a concentration of 2.5 mg/mL. This concentration was chosen since at lower concentration the cloud point is more difficult to detect, because less polymer chains will aggregate during the phase transition at lower concentrations resulting in the formation of smaller precipitated particles that scatter less light. Figure 2 clearly demonstrates that at low temperatures the polymer solution has close to 100% transmittance indicative of a clear polymer solution. Upon increasing the temperature, the polymer chains precipitate resulting in the formation of large aggregates that scatter away the light as indicated by 0% transmittance. The difference in the turbidity curves during first and second cooling are due to the influence of the history of the solid polymer particles that is still present in the first cooling run while this history is erased during complete dissolution in the first heating run. The temperature at 50% transmission represents the cloud point (CP) of the polymer solution, which is 18.3 °C for both heating runs. During these heating runs, the solubility of the polymer at temperatures below the CP is based on the formation of a large number of hydrogen bonds between the repeating units of the polymer chain (ethylene glycol units) and water molecules that form a hydration shell. Upon increasing the temperature the hydrogen bonds are weakened and finally broken resulting in the loss of the hydration shell leaving the non-hydrated hydrophobic polymer chains behind. At the CP, the polymer chain collapses and the water is released into the bulk water. This polymer phase transition is driven by the increase of entropy of releasing the water molecules into the bulk water. Below the CP, the polymers are well solvated and, thus, are exposed to the polar aqueous environment while in the precipitated state above the CP the polymer globule is less polar. This polarity transition around the polymer chains is the basis for the sensing ability of the poly(DEGMA-*stat*-PyMMA) copolymer **5** that will be discussed in the following.

**Figure 3.**
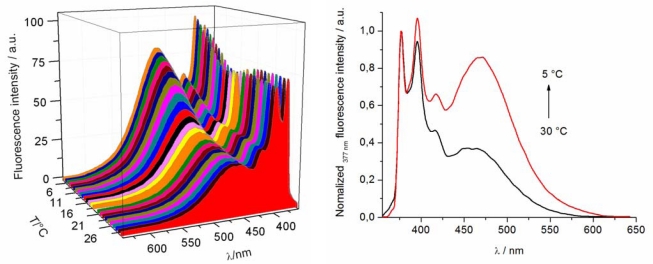
(**a**) Waterfall plot of the fluorescence spectra as a function of temperature (excitation wavelength 342 nm) and (**b**) normalized fluorescence intensity (377 nm) at 5 °C (red) and 30 °C (black) of a solution of pyrene-labeled copolymer **5** in water at 1.0 mg mL^−1^.

The temperature sensing ability of the pyrene-labeled copolymer **5** in aqueous solution was investigated by temperature controlled fluorescence spectroscopy at a polymer concentration of 1 mg/mL. Figure 3a shows the resulting waterfall plot of the fluorescence spectra recorded in between 5 °C to 30 °C (λ_exc_ = 342 nm; one spectra per degree). Three characteristic pyrene emission bands are observed at 467 nm (broad), 377 nm and 395 nm, which are assigned to the excimer emission (I_E_ > 450 nm) and the individual pyrene molecule emission (I_1_ at 377 nm and I_5_ at 395 nm) of the pyrene units in the copolymer. This 3D representation clearly demonstrates that the intensity of I_E_ at 467 nm increases with decreasing temperature, along with a small red shift of the excimer emission from 462 to 471 nm (Figure 3b).

The stronger excimer emission at lower temperatures can be related to the high polarity of the aqueous environment, which enhances the hydrophobic association of individual pyrene molecules. This association is further facilitated by the high mobility of the hydrated polymer chains in solution. At higher temperatures, in the precipitated state, the polarity around the pyrene molecules is lower compared to the hydrated state lowering the formation of excimers. In addition, the increased microviscosity in the collapsed polymer globules might also hinder the excimer formation.

The ratio of I_5_/I_1_ (I_5_ at 395 nm and I_1_ at 377 nm) is 1.07 at 5 °C, which is quite similar to the value in ethanol of 1.06 indicating that the pyrene molecules are not fully exposed to the solvent, *i.e.*, they apparently form hydrophobic clusters as is also indicated by the excimer emission. This I_5_/I_1_ ratio is linearly decreasing during the polymer phase transition upon heating and finally reaches a value of 0.94 at 30 °C, which is similar to the value of acidic acid of 0.95 [36], surprisingly indicating a more polar environment in the precipitated state. This increased polarity is most likely due to breaking of the hydrophobic pyrene clusters and, therefore, the pyrene groups can interact (e.g., dipole-dipole) with the polar ethylene oxide side chains of the polymer chain.

**Figure 4.**
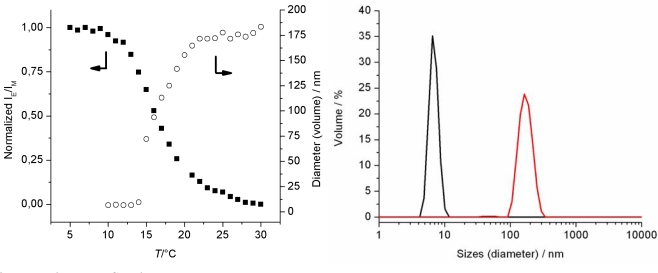
(**a**) The ratio of excimer (467 nm) to monomer (395 nm) emission intensities (I_E_/I_M_, black squares) and the hydrodynamic radius of the polymer globules (DLS, open circles) of copolymer **5** at 1.0 mg mL^−1^ as function of temperature. (**b**) The hydrodynamic radius of the polymer globules at 5 °C (black) and 30 °C (red) as determined by DLS at 1.0 mg mL^−1^ (CONTIN routine).

To quantify the sensing ability of the pyrene-labeled copolymer **5**, the normalized ratio of excimer emission to monomer emission intensities (I_E_/I_M_) *versus* temperature was investigated since this ratio is expected to be independent from fluctuations in polymer concentration making the read-out of the sensor more robust (Figure 4a). Three distinct regimes are present in I_E_/I_M_ *versus* temperature plot. Below 10 °C when the polymer is fully soluble in aqueous solution, the I_E_/I_M_ ratio is constant. A strong decrease in I_E_/I_M_ is observed upon increasing the temperature from 11 °C and 21 °C, which can be regarded as the temperature sensing regime of copolymer **5**. Finally, above 21 °C the I_E_/I_M_ ratio only shows a minor decrease, which might be attributed to increased chain mobility within the precipitated polymer globules rather than a polymer phase transition. The close similarity of the I_E_/I_M_ ratio and the turbidimetry results clearly demonstrates that indeed the polymer phase transition can be employed for the development of a fluorescent thermometer.

The temperature induced phase transition of copolymer **5** was investigated in further detail by dynamic light scattering as a function of temperature (DLS; Figure 4). Representative size distributions of the polymer globules at temperatures below and above the phase transition are illustrated in Figure 4b (CONTIN analysis). Below the CP at 10 °C the polymer globule has a hydrodynamic radius of 7 nm corresponding to individual hydrated polymer chains. Above the CP at 30 °C where polymer chains are precipitated, larger aggregates with a hydrodynamic radius of 183 nm are observed. Apparently, the polymer concentration is low enough to prevent further aggregation into micrometer sized particles. It is rather surprising that the precipitated polymer aggregates have a narrow size distribution with a PDI_particle_ value of 0.060 (obtained from the Cumulants analysis).

The hydrodynamic radius of the polymer globules is plotted as a function of temperature in Figure 4a, revealing a very similar temperature transition as previously observed by turbidimetry investigations as well as the I_E_/I_M_ ratio. From 10 to 14 °C the particle size is constant around 7 nm, indicating the presence of individual hydrated polymer chains in solution. A further increase in temperature results in a strong increase in the diameter of the polymer globules indicative of a temperature induced aggregation. The demixing point of the polymer solution might be regarded to be 15 °C where the Z average diameter of the aggregates is already 78 nm with a PDI_particle_ of 0.222 (Cumulant analysis). In between 16 and 20 °C the polymer chains are further dehydrated making them more hydrophobic resulting in further aggregation of the initial aggregates as evidenced by the increase of the hydrodynamic radius. Heating beyond the polymer phase transition, *i.e.*, above 21 °C, does not further affect the size of the aggregates. These DLS results are in very good agreement with the sensing behavior of the copolymer confirming that the change in pyrene emission is based on the temperature induced polymer phase transition.

## 3. Experimental Section

### 3.1. Materials

Di(ethylene glycol) methylether methacrylate (DEGMA) was purchased from Sigma-Aldrich and was purified with an inhibitor-remover before use. Pyrene-1-methanol was purchased from Sigma-Aldrich and was used without purification. Azobis(isobutyronitrile) (AIBN, Aldrich) was recrystallized from methanol prior to use. 2-Cyano-2-butyl dithiobenzoate (CBDB) [38] was prepared according to a literature procedure for a related compound. All analytical grade solvents were purchased from Biosolve Ltd. or Fluka. The deuterated solvents (CDCl_3_ or CD_2_Cl_2_) for NMR spectroscopy were obtained from Cambridge Isotope Laboratories.

### 3.2. Instrumentation

Size-exclusion chromatography (SEC) was performed on a Shimadzu system equipped with a SCL-10A system controller, a LC-10AD pump, a RID-10A refractive index detector, a SPD-10A UV detector at 290 nm and a PSS SDV column with chloroform-triethylamine-2-propanol (94:4:2) as eluent and the column oven was set to 50 °C (polystyrene calibration). Poly(styrene) (PS) samples were used as calibration standards.

Nuclear magnetic resonance spectra were recorded on a Varian Mercury 400 MHz spectrometer at 298 K. Chemical shifts are reported in parts per million (ppm) calibrated to an internal standard, tetramethylsilane (TMS) in deuterated solvents (CDCl_3_ or CD_2_Cl_2_).

UV/vis spectra were recorded on a Perkin-Elmer Lamda-45 UV/vis spectrophotometer. For fluorescence measurements a Perkin-Elmer Luminescence Spectrometer LS 50B with a PTP-1 Peltier Temperature Programmer were used. For the temperature measurements on these spectrophotometers a temperature profile with a temperature rate of 0.5 °C/min was used.

Elemental analyses were carried out on a EuroVector EuroEA300 elemental analyzer for CHNSO. The cloud point measurements for the identification of the LCST behavior were performed by heating the polymer (2.5 mg/mL) in water from 0 to 105 °C with a heating rate of 1.0 °C per minute followed by cooling to 0 °C at a cooling rate of 1.0 °C per minute after keeping it 10 minutes at 105 °C. This cycle was repeated two times. During these controlled cycles the transmission through the solutions was monitored in a Crystal16™ from Avantium Technologies. The cloud points are reported as the 50% transmittance temperature in the second heating run.

Dynamic light scattering (DLS) measurements were carried out on a Zetasizer Nano ZS (Malvern Instruments, Malvern, U.K.) operating with a laser beam at 633 nm and a scattering angle of 173°. The polymer was dissolved in water (1.0 mg/mL) and transferred into a quartz cuvette. The DLS measurements were performed between 10 and 30 °C using steps of 1 °C and an equilibrium time of 120 seconds. The solution was measured three times for 60 seconds at every temperature. The mean particle size was approximated as the effective (Z average) diameter and the width of the distribution as the polydispersity index (PDI_particle_) that was obtained by the Cumulants method assuming a spherical shape. Furthermore, the particle size distribution was calculated applying the NNLS mode (CONTIN routine).

### 3.3. Synthesis of pyrene-1-ylmethyl-methacrylate (PyMMA) monomer (**3**)

To a solution of triethylamine (1.80 mL, 12.8 mmol) in anhydrous THF (50 mL), pyrene-1-ylmethanol (1.0 g, 4.3 mmol) was added. Methacryloyl chloride (1.24 mL, 12.8 mmol) was added dropwise to this clear solution at 0 °C. The reaction was then stirred at room temperature overnight (24 hours total reaction time). Subsequently, the reaction medium was filtered and the solvent was evaporated under reduced pressure. Afterwards the solid residue was dissolved in diethyl ether and washed with water. After evaporation of the diethyl ether, the crude monomer was purified by recrystallization from ethanol at 40 °C. Yield: 40%. GC-MS: m/z (%) = 300 (38) [M^+^], 215 (100) [M^+^ –C_4_H_5_O_2_], 203 (9) [C_6_H_10_+ H^+^], 189 (8), 107 (5), 94 (9), 41 (10) [all aromatic fragmentation]. ^1^H-NMR (400 MHz, CDCl_3_): *δ* = 8.32–8.00 (m, 9H, aromatic H^2–10^), 6.16 (s, 1H, H^12^), 5.91 (s, 2H, H^11^), 5.57 (s, 1H, H^12^), 1.98 (s, 3H, H^13^) ppm. ^13^C-NMR (100 MHz, CDCl_3_): *δ* = 167.4 (C=O), 136.2, 131.7, 131.2, 130.7, 129.5, 129.0, 128.1, 127.8, 127.6, 127.3, 126.1, 126.0, 125.5, 125.4, 124.9, 124.6, 124.5, 122.9 (16 aromatic C and –C=CH_2_), 65.0 (–CH_2_-O), 18.4 (–CH_3_) ppm. Elemental analysis: C_21_H_16_O_2_ (300.35): cal.: C 83.98% H 5.37%; found: C 84.08% H 5.64%. UV/vis (*n*-heptane): *λ*_max_/nm (*ε*/(M^−1^·cm^−1^): 201 (16,120), 233 (26,060), 242 (46,680), 265 (16,350), 276 (31,270), 312 (7,760), 326 (20,210), 342 (32,520). Fluorescence (THF): *λ*_max_/nm: 377, 394, 416 (shoulder), 435 (shoulder).

### 3.4. Synthesis of poly(DEGMA-stat-PyMMA) copolymer **5**

Poly(MMA-*stat*-PyMMA) was prepared in a closed reaction vessel with a [DEGMA]:[PyMMA]: [CBDB]:[AIBN] ratio of 95:5:1:0.25. AIBN (1.64 mg, 0.01 mmol), PyMMA (60.1 mg, 0.2 mmol) and CBDB (9.41 mg, 0.04 mmol) were dissolved in a solution of DEGMA (0.70 mL, 3.8 mmol) and toluene (1.30 mL). Before the polymerization, the solution was degassed with argon for 30 min. The reaction mixture was heated to 70 °C for 12 hours. Afterwards the polymer mixture was diluted with dichloromethane and precipitated twice in *n*-hexane resulting in a pink viscous oil. ^1^H-NMR (400 MHz, CD_2_Cl_2_): *δ* = 8.51–8.02 (m, 46H, H pyrene), 7.89 (m, 1H, H RAFT-agent), 7.56 (m, 1H, H RAFT-agent), 7.39 (m, 2H, H RAFT-agent), 5.76 (s, 8H, –CH_2_-O–), 4.10 (s, 175H, –CH_2_-O–), 3.67, 3.61 and 3.53 (s, 568H, –CH_2_-CH_2_–), 3.36 (s, 297H, –O-CH_3_), 1.98–0.88 (m, 500H, -CH_2_– and -CH_3_) ppm. UV/vis (1,4-dioxane): *λ*_max_/nm [*ε*/(M^−1^·cm^−1^)]: 244 (369,580), 266 (183,240), 277 (337,030), 314 (90,630), 328 (214,340), 342 (316,000). Fluorescence (H_2_O): *λ*_max_/nm: 377, 395, 467. Select characterization and composition data are given in Tables 1 and 2.

## 4. Conclusions

A well-defined fluorescent thermoresponsive copolymer based on poly(DEGMA) side-chain functionalized with pyrene has been synthesized by RAFT polymerization. It could demonstrated by temperature controlled fluorescence investigations that this polymer acts as a soluble fluorescent temperature sensor in water. At temperatures below the polymer phase transition, the polymer chains are hydrated as demonstrated by DLS and, thus, the pyrene molecules are exposed to the polar aqueous environment driving excimer formation. Above the LCST phase transition of the polymer, the polymer chains are dehydrated and demix from the aqueous solution providing a less polar environment for the pyrene inside the polymer aggregates. During the phase transition, a gradual decrease in I_E_/I_M_ ratio is observed, which can be used to detect the temperature of the solution in between 11 °C and 21 °C, *i.e.*, the temperature sensing regime. Interpretation of the I_E_/I_M_ ratio as sensing signal is believed to make the sensor more robust compared to looking at individual emission intensities since it will be less dependent on polymer concentration. Turbidimetry and DLS demonstrated that the polymer phase transition also occurred in the observed temperature sensing regime confirming that indeed the polymer phase transition induces the change in I_E_/I_M_ ratio of the attached pyrene molecules.

## Figures and Tables

**Table 1.** Selected characterization data of the precipitated poly(DEGMA-*stat*-PyMMA).

**Sample**	**Ratio n/m**	**Yield [mg]**	**M_n_ [g/mol]^a^**	**PDI^a^**
**5**	95/5	551	29,000	1.20

a Obtained from SEC (RI) using CHCl_3_ eluent and PS standards.

**Table 2.** Composition of poly(DEGMA-*stat*-PyMMA).

**Sample**	**Theo. ratio DEGMA/PyMMA n/m**	**Composition^a^^1^H-NMR signal [%]**	**Composition^b^ UV/vis pyrene [%]**
**5**	95/5	4.5	5.0

a Obtained from the proton integrals of the pyrene and backbone using ^1^H-NMR spectroscopy;

b Obtained from the ε of the UV/vis spectrum using the Lambert-Beer-Law and Mn of SEC.
